# The effect of the HMGB1/RAGE/TLR4/NF‐κB signalling pathway in patients with idiopathic epilepsy and its relationship with toxoplasmosis

**DOI:** 10.1111/jcmm.18542

**Published:** 2024-07-24

**Authors:** Hayriye Soytürk, Cansu Önal, Ümit Kılıç, Şule Aydın Türkoğlu, Erol Ayaz

**Affiliations:** ^1^ Bolu Abant Izzet Baysal University, Institute of Graduate Studies Interdisciplinary Neuroscience Bolu Turkey; ^2^ Zonguldak Bülent Ecevit University Department of Molecular Biology and Genetics, Faculty of Science Zonguldak Turkey; ^3^ Duzce University Vocational School of Health Services Duzce Turkey; ^4^ Department of Neurology, Faculty of Medicine Bolu Abant Izzet Baysal University Bolu Turkey; ^5^ Department of Parasitology, Faculty of Medicine Bolu Abant Izzet Baysal University Bolu Turkey

**Keywords:** epilepsy, gene expression, HMGB/RAGE/TLR4/NF‐κB signalling pathway, inflammation, Q‐PCR, toxoplasmosis

## Abstract

This study aims to investigate the relationship between toxoplasmosis and this pathway, which may be effective in the formation of epilepsy by acting through the HMGB1/RAGE/TLR4/NF‐κB signalling pathway in patients with idiopathic epilepsy. In the study, four different experimental groups were formed by selecting *Toxoplasma gondii* IgG positive and negative patients with idiopathic epilepsy and healthy controls. Experimental groups were as follows: Group 1: Epilepsy+/Toxo− (E+, T−) (*n* = 10), Group 2: Epilepsy−/Toxo− (E−, T−) (*n* = 10), Group 3: Epilepsy−/Toxo+ (E−, T+) (*n* = 10), Group 4: Epilepsy+/Toxo+ (E+, T+) (*n* = 10). HMGB1, RAGE, TLR4, TLR1, TLR2, TLR3, IRAK1, IRAK2, IKBKB, IKBKG, BCL3, IL1β, IL10, 1 L8 and TNFα mRNA expression levels in the HMGB/RAGE/TLR4/NF‐κB signalling pathway were determined by quantitative simultaneous PCR (qRT‐PCR) after collecting blood samples from all patients in the groups. Statistical analysis was performed by one‐way ANOVA followed by LSD post‐hoc tests, and *p* < 0.05 was considered to denote statistical significance. The gene expression levels of HMGB1, TLR4, IL10, IL1B, IL8, and TLR2 were significantly higher in the G1 group than in the other groups (*p* < 0.05). In the G3 group, RAGE and BCL3 gene expression levels were significantly higher than in the other groups (*p* < 0.05). In the G4 group, however, IRAK2, IKBKB, and IKBKG gene expression levels were significantly higher than in the other groups (*p* < 0.05). HMGB1, TLR4, IRAK2, IKBKB, IL10, IL1B, IL1B, and IL8 in this signalling pathway are highly expressed in epilepsy patients in G1 and seizures occur with the stimulation of excitatory mechanisms by acting through this pathway. The signalling pathway in epilepsy may be activated by HMGB1, TLR4, and TLR2, which are considered to increase the level of proinflammatory cytokines. In *T. gondii*, this pathway is activated by RAGE and BCL3.

## 1 INTRODUCTION

Epilepsy is a neurological disorder characterized by recurrent epileptic seizures, which affects approximately 60 million people worldwide.^1^ Seizures are known to be caused by the synchronized firing of overexcited neurons. Based on this knowledge, current antiepileptic drugs are used to control seizures by enhancing inhibitory mechanisms or blocking excitatory mechanisms. Despite all these treatment strategies, 30% of epilepsy patients are drug‐resistant.^2^ Recurrent and unpredictable seizures not only affect the patient's brain function, causing mental and cognitive impairment but can also be life‐threatening.^3^, ^4^, ^5^, ^6^ Previous studies have shown that the electrical activity of neurons can be affected by inflammatory factors, thereby regulating the excitability of the central nervous system (CNS).^7^, ^8^ Conversely, seizures can induce inflammatory responses that cause damage to the CNS, which may be one of the pathological underpinnings of intractable epilepsy.^9^ Although the mechanisms of epilepsy are complex, many well‐defined and recognized mechanisms have shed light on the understanding of epilepsy. In recent years, the neuroinflammatory response has been added to these mechanisms. Inflammatory factors, in addition to causing local inflammatory responses, can affect the electrical activity of neurons and glial cells; epileptic seizures can also cause neuroinflammatory responses, further aggravating neuronal damage, so that the conditions in which inflammation and epileptic seizures influence each other continue to increase, worsen, and lead to a vicious cycle.^9^, ^10^

Parasitic infections are among the leading causes of epileptic seizures or epilepsy, as well as neurological and mental health conditions.^11^, ^12^ *Toxoplasma gondii* (*T. gondii*) infection is reported to affect one‐third of the world's population, particularly in low‐ and middle‐income countries.^13^, ^14^ Present data strongly suggest a possible association between toxoplasmosis and epilepsy,^15^ but there are also results suggesting the opposite.^16^

Many parasitic, bacterial and viral pathogens are known to negatively affect the nuclear factor kappa B (NF‐κB) pathway for their survival. To modulate host cells, Toxoplasma strains secrete specialized effector proteins that activate the NF‐κB pathway, which plays an important role in cell death, immunity and inflammation. NF‐κB pathways control numerous NF‐κB‐regulated genes that are associated with many diseases. The toll‐like receptor (TLR) or interleukin‐1 (IL‐1) receptor (IL‐1R) family functions as extracellular sensors through their common protein domains to detect pathogens and cytotoxic molecules and enable cells to mount effective neuroprotective immune responses. IRAK‐1 of the interleukin‐1 receptor‐associated kinases (IRAK) family has been associated with TLR/IL‐1R (TIR) signalling. B‐cell leukaemia/lymphoma 3 (Bcl‐3) is believed to be required for the activity of adaptive immune cells. Bcl‐3 dysregulation has been observed in several autoimmune diseases, and Bcl3‐deficient animals are more susceptible to bacterial and parasitic infections. In addition, neuroglial cells in the CNS initiate and regulate the expression of NF‐κB transcription factor, during inflammation and various inflammatory processes implicated in the pathology of several neurodegenerative diseases. It has also been reported that some parasites, such as *T. gondii*, are involved in the neuropathological processes of seizures and epilepsy via the NF‐κB pathway, which plays a role in the immune and inflammatory response.

High mobility group box 1 (HMGB1), which is also an inflammatory protein, attracts attention in epilepsy studies conducted with animal models. HMGB1 levels were found to be higher in the blood of animals with active epilepsy compared to healthy subjects or controls.^17^ A clinical study showed that HMGB1 levels are proportional to the severity of epilepsy and that high HMGB1 levels may represent an increased likelihood of antiepileptic drug resistance.^18^ It has also been reported that seizure frequency can be predicted by serum HMGB1 levels.^19^

HMGB1 can be actively or passively released from neurons and glial cells into the extracellular space. In the extracellular space, HMGB1 is slightly oxidized to form disulfide HMGB1 with a disulfide bond between Cys23 and Cys45, but Cys106 remains in the reduced form.^20^ The disulfide HMGB1 binds to TLR4 to initiate a neuroinflammatory response.^20^, ^21^ TLRs were first identified in *Drosophila melanogaster* in 1988, followed by the identified human homologue TLR4 in 1997. To date, 13 different TLR species (TLR1‐13) have been discovered in mammals, 10 of which are present in humans (TLR1‐10) and contain both intracellular and extracellular segments.^22^ These are members of the family of receptors that bind to pathogen‐associated molecular patterns to initiate innate immune responses. They are widely expressed in microglia, astrocytes and CNS neurons.^23^ TLR4 has been associated with intracellular, transmembrane and extracellular domains.^24^ The major functions of TLR4 are involved in the regulation of cytokine secretion and microglial phagocytic activity. TLR4 signalling in the brain directs autoimmune responses and initiates neuroinflammation, which plays an important role in several brain disorders (e.g. cerebrovascular disease, brain tumours and epilepsy).^25^ HMGB1 is mainly involved in the epilepsy pathophysiology by interacting with the primary receptor TLR4. HMGB1 can translocate to the cytosol, plasma membrane and extracellular space in response to various stressors. When neurons, glial cells and immune cells are stimulated by inflammatory factors (e.g. IL‐1β and TNF‐α) or activated in response to oxidative stress, HMGB1 is actively released from the intracellular to the extracellular space.

Extracellularly released HMGB1 binds to TLR4 and receptors for advanced glycation end‐products (RAGE) on the surface of glial cells and neurons. The activated HMGB1/TLR4 signalling pathway transduces signals through myeloid differentiation factor 88 (MyD88) dependent and independent pathways, and stimulates nuclear factor κB (NF‐κB) translocation into the nucleus by transcription of target genes responsible for the neuroimmune inflammatory response.^26^, ^27^ Upon activation by HMGB1/TLR4 signalling, phosphorylation of the NR2B subunit of the N‐methyl‐D‐aspartic acid (NMDA) receptor leads to Ca^2+^ influx, which makes neuronal cells hyperexcitable and initiates epileptogenesis.^28^, ^29^

Many studies have shown that chronic *T. gondii* infection in the brain is associated with changes in neuronal structure, neurochemistry and behavioural changes.^30^ *T. gondii* infection is also associated with neuropsychiatric disorders and epilepsy.^31^, ^32^, ^33^ High levels of *T. gondii* seropositivity and high levels of *T. gondii* antibodies have been found in patients with cryptogenic epilepsy.^34^, ^35^ Numerous pro‐inflammatory factors have been found in the serum of hosts infected by *T. gondii*, including tumour necrosis factor‐alpha (TNF‐α), interferon‐gamma and nitric oxide (NO).^36^, ^37^

Extracellularly released HMGB1 is a weapon in the fight against infection through proinflammatory response and immune regulation. *T. gondii* leads to inflammatory pathological changes during a chronic infection. To investigate whether HMGB1 contributes to toxoplasmosis lesions, HMGB1 changes during *T. gondii* infection were investigated with results showing that HMGB1 transcription was downregulated in mouse macrophage antinuclear antibody (Ana‐1) cell line and mouse peritoneal macrophages after *T. gondii* vaccination, but upregulated in IFN‐y treated macrophages and intraperitoneal exudate cells of *T. gondii*.

In this study, it was predicted that *T. gondii* may cause epilepsy in patients with idiopathic epilepsy by acting through the NF‐κB signalling pathway and it was aimed to determine the gene expression levels of HMGB1, RAGE, TLR4, TLR1, TLR2, TLR3, IRAK1, IRAK2, IKBKB, IKBKG, BCL3, IL1β, IL10, 1 L8 and TNFα associated with this signalling pathway. Gene expression levels of the HMGB1/RAGE/TLR4/NF‐κB signalling pathway, which is effective in both epilepsy and toxoplasmosis, were determined in patients with idiopathic epilepsy.

## 2 MATERIALS AND METHODS

This study was conducted at the Department of Neurology, Abant İzzet Baysal University Faculty of Medicine in 2020. The study was approved by the local ethics committee (protocol number 2016/213) and informed consent was obtained from all participants. The Declaration of Helsinki was adhered to during the study period and writing of the manuscript. The study was supported by TUBITAK 3001—Startup R&D with project number 317S052.

### 2.1 Group formation and patient sample collection

**FIGURE 1:**
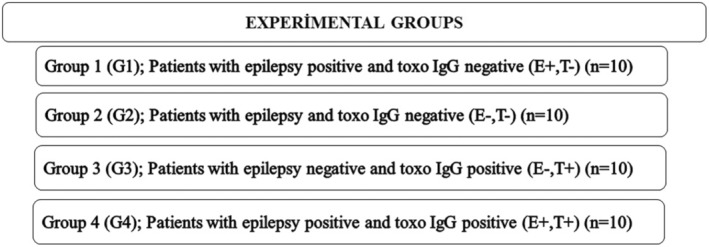
Experimental groups.

A balanced gender and age distribution was provided for a statistically significant evaluation of the sociodemographic characteristics of each group. *T. gondii* IgG positive and negative individuals were selected. In line with these characteristics, four different study groups were formed. Blood samples were taken from all patients in these groups (Figure 1). Once the blood was collected, the samples were stored at −80°C until RNA isolation.

#### 2.1.1 Q‐PCR method

Total mRNA was isolated, cDNA synthesis was performed, and quantitative RT‐PCR (qRT‐PCR) experiments were performed to detect changes in gene expression levels. RNA isolation: For RNA isolation from tissue samples, 1 mL of Trizol solution was added to a 50 mg tissue sample and homogenized. The tubes were incubated at room temperature (RT) for 5 min, then 200 μL of chloroform was added and the mixture was manually vortexed for 15 s. The tubes were held at RT for 3 min and centrifuged at 12,000*g* for 15 min at 4°C. The clear upper phase was transferred to a new tube and 500 μL of 100% isopropanol was added. After incubation at RT for 10 min, the tubes were centrifuged at 12,000*g* for 10 min at 4°C. At this time, a white precipitate formed at the bottom of the tube due to RNA in the sample. The liquid was carefully removed without disturbing the precipitate and the RNA was washed with 1 mL of 75% ethanol. After centrifugation at 7500*g* for 5 min at 4°C, the resulting RNA was dissolved in 20–50 μL of DEPC‐ddH_2_O and its concentration was measured.

#### 2.1.2 cDNA synthesis

For cDNA synthesis, 1 μg RNA was mixed with 2 μL oligo dT and DEPC‐ddH_2_O in a final volume of 8 μL. The mixture was incubated for 5 min at 70°C. Then, 10 μL of 2X reaction buffer and 2 μL of reverse transcriptase enzyme were added and samples were incubated at 42°C for 1 h followed by 5 min at 80°C. The resulting cDNA samples were stored at −20°C.

#### 2.1.3 qRT‐PCR

Primers were designed with high specificity to bind to the target gene regions for qRT‐PCR experiments. The Amplify program was used to design and study the properties of the oligos, such as melting temperature (Tm) and primary dimer formation. To ensure specificity, the primers were selected from the exon‐intron junction regions to avoid binding to other regions in the genome. Primer specificity was confirmed by in silico PCR using the UC Genome Browser. To measure mRNA expression levels, each qRT‐PCR reaction contained 1 μL of cDNA, 1 μL of primer mixture (10 μM, forward+reverse), 10 μL of 2X SYBR Green, and 8 μL of ddH_2_O. The following program was followed for the reaction: 95°C for 5 min, [95°C for 15 s, 60°C for 30 s, 72°C for 30 s] × 40, 72°C for 5 min (Table 1).

**TABLE 1:** Primers.

HUMAN	Primers (5′–3′)	Tm (°C)
HMGB1‐F	TATGGCAAAAGCGGACAAGG	58
RAGE‐F	GTGTCCTTCCCAACGGCTC	57
IL1B‐F	GGAGAATGACCTGAGCACCT	59
IL8‐F	TTCAGAGACAGCAGAGCACA	57
TLR1‐F	AACTCTGCTGATCGTCACCA	57
TLR2‐F	TGGATGCAGAACCCATGGAT	57
TLR3‐F	GGAGGAGTTTTCGATGCCAC	59
TLR4‐F	CCAGCCTCCTCAGAAACAGA	59
TNF‐F	AGGACCAGCTAAGAGGGAGA	59
IRAK1‐F	CCCCTTCTTCTACCAAGCCA	59
IRAK2‐F	CAGCTGCGGAAGATCAAGTC	59
IKBKB‐F	TGTGTGAGACTCCTGCGATT	57
IKBKG‐F	TCTGTCTGCTCGAACCACTT	57
BCL3‐F	CAGTGGACATTAAGAGCGC	59
IL10‐F	GCCAAGCCTTGTCTGAGATG	59
Human Beta Actin‐F	CATGGAATCCTGTGGCATCC	59

#### 2.1.4 Analysis of qRT‐PCR results

To avoid inter‐sample differences and possible pipetting errors in the detection of mRNA expression levels, normalization was performed using a housekeeping gene, β‐actin. The analysis was performed using the ddCt31 method and the following equation: ddCt = Ct (target gene) Ct (housekeeping gene). The target gene expression was calculated as 2^(−ddCt)^.

## 3 RESULTS

The gene expression levels of HMGB1, RAGE, IL1β, IL8, IL1β, IRAK1, IRAK2, IKBKB, IKBKG, TLR1, TLR2, TLR3, TLR4 and TNFα were evaluated using the SPSS software. Statistical analysis was performed using the one‐way analysis of variance to determine whether there were differences between groups. The post hoc test was used to determine which group was responsible for the difference. The LSD test was used as a post hoc test, and differences with a *p*‐value of 0.05 were considered significant.

**FIGURE 2:**
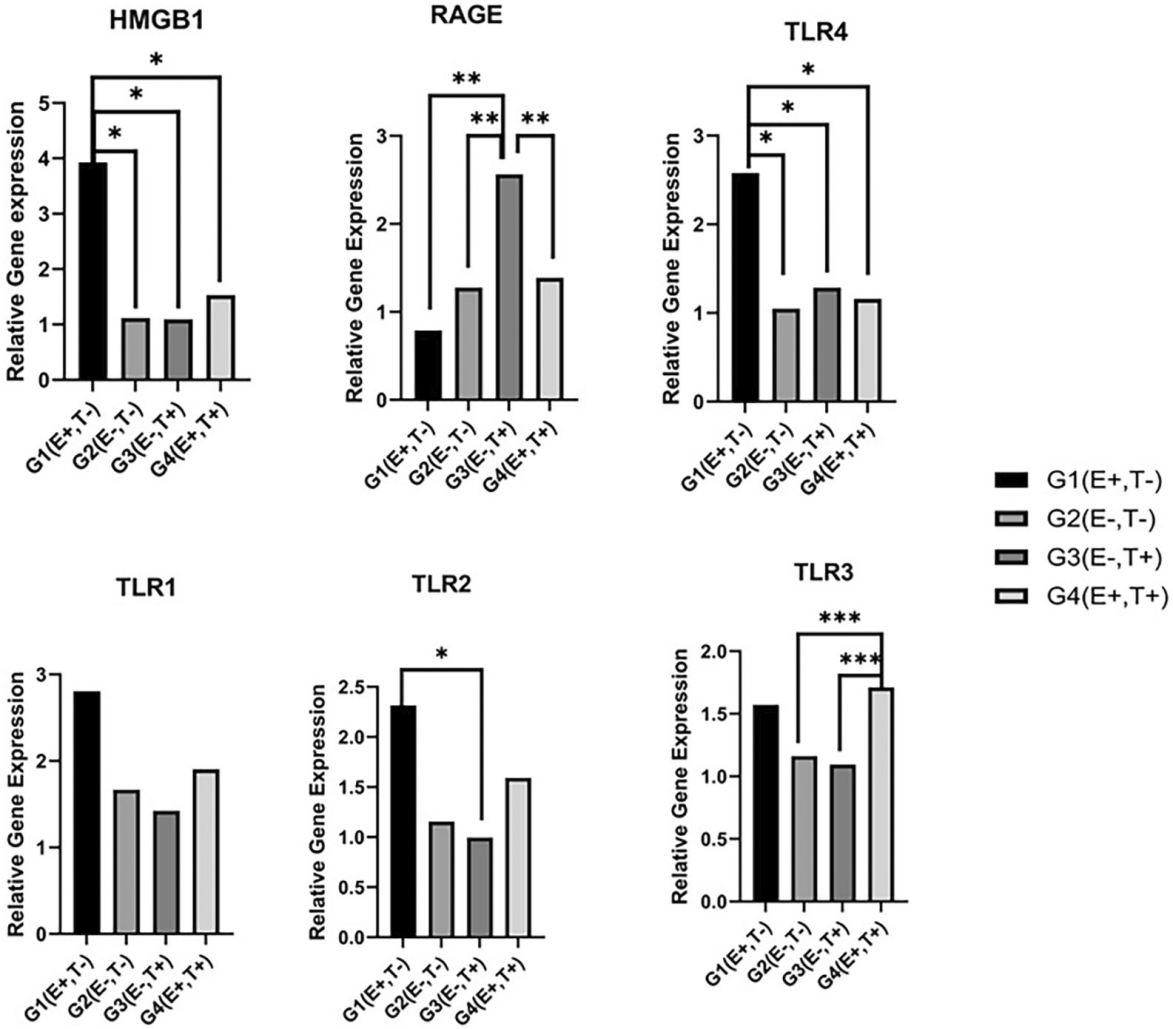
Relative gene expression levels of HMGB1, RAGE, TLR4, TLR2, TLR3 and TLR1. HMGB1 gene expression level in G1 (E+, T−) group. *G1 is significantly different from other groups (*p* < 0.05). **G3 is significantly different from other groups; ***G4 is significantly different from other groups.

The HMGB1 gene expression level was found to be statistically significantly higher in the G1 (E+, T−) group than in the G2 (E−, T−), G3 (E−, T+) and G4 (E+, T+) groups (*p* < 0.05). HMGB1 was expressed 3.9‐fold higher in G1, 1.1‐fold higher in G2, 1.1‐fold higher in G3 and 1.4‐fold higher in G4 (Figure 2).

RAGE gene expression level in G3 (E−, T+) group was statistically significantly higher than in G1 (E+, T−), G2 (E−, T−), and G4 (E+, T+) groups (*p* < 0.05). RAGE was expressed 0.8‐fold more in the G1 group, 1.3‐fold more in the G2 group, 2.6‐fold more in the G3 group, and 1.4‐fold more in the G4 group (Figure 2).

TLR4 gene expression level in G1 (E+, T−) group was significantly higher than in G2 (E−, T−), G3 (E−, T+), and G4 (E+, T+) groups (*p* < 0.05). TLR4 was expressed 2.6‐fold more in the G1 group, 1‐fold more in the G2 group, 1.3‐fold more in the G3 group, and 1.2‐fold more in the G4 group (*p* < 0.05).

TLR2 gene expression level was significantly higher in the G1 (E+, T−) group than in the G3 (E−, T+) group (*p* < 0.05). TLR2 was expressed 2.3‐fold more in the G1 group, 1.1‐fold more in the G2 group, 1‐fold more in the G3 group, and 1.6‐fold more in the G4 group (Figure 2).

TLR3 gene expression level in G4 (E+, T+) group was significantly higher than in G2 (E−, T−), and G3 (E−, T+) groups (*p* < 0.05). TLR3 was expressed 1.6‐fold higher in the G1 group, 1.2‐fold higher in the G2 group, 1.1‐fold higher in the G3 group, and 1.7‐fold higher in the G4 group (Figure 2).

For TLR3, there was no significant difference between all groups, but it was least expressed in the *T. gondii* group and highest in both the epilepsy and toxo‐positive groups (Figure 2).

When TLR1 gene expression levels were evaluated, there was no significant difference between groups (*p* > 0.05). TLR1 was expressed 2.8 times higher in the G1 group, 1.7 times higher in the G2 group, 1.4 times higher in the G3 group, and 1.9 times higher in the G4 group (Figure 2).

**FIGURE 3:**
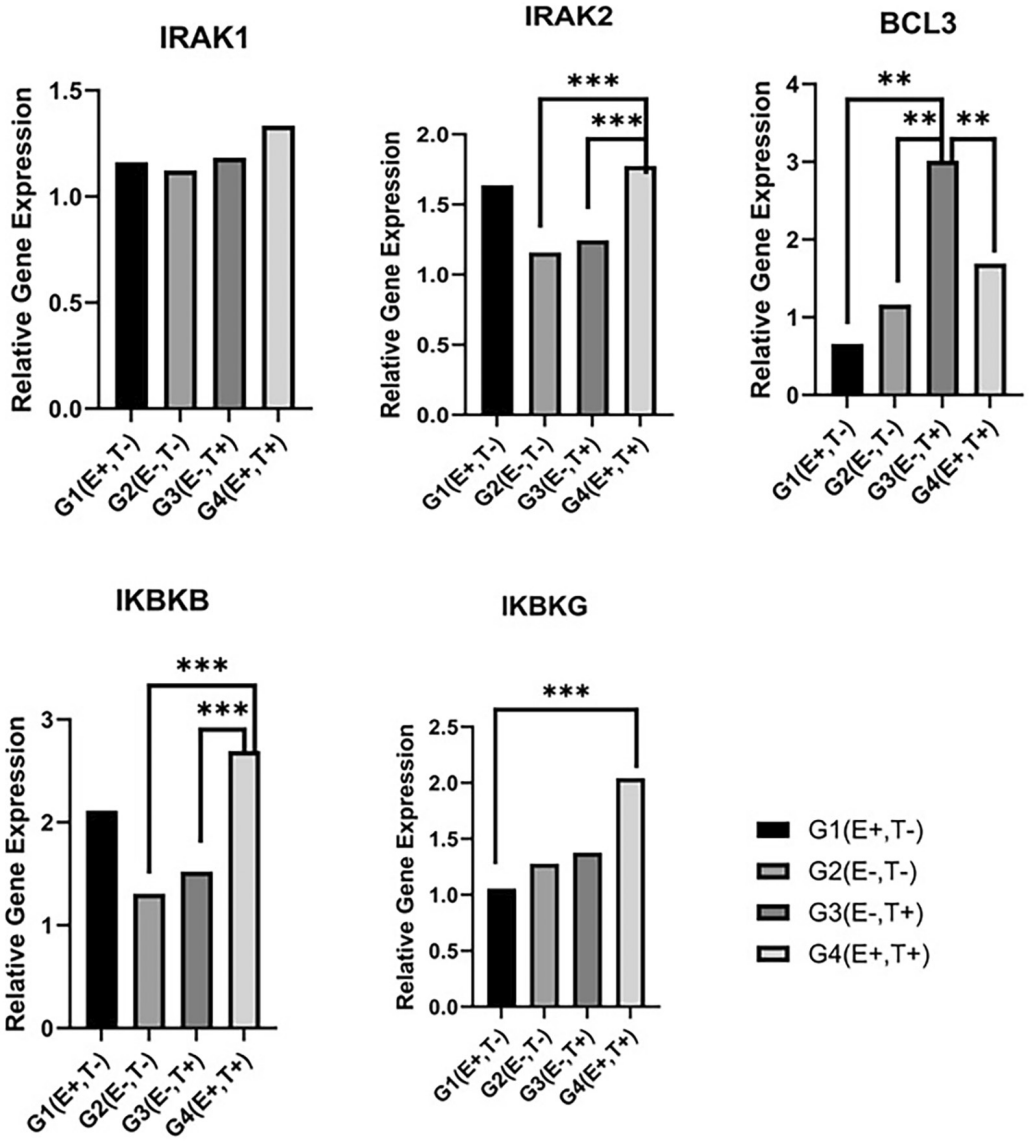
Relative gene expression levels of IRAK1, IRAK2, BCL3, IKBKB and IKBKG. HMGB1 gene expression level in G1 (E+, T−) group.   **G3 was significantly different from other groups; ***G4 was significantly different from other groups.

There is no significant difference between the groups in terms of IRAK1 gene expression levels. IRAK1 was expressed 1.2‐fold higher in G1 (E+, T−), 1.1‐fold higher in G2 (E−, T−), 1.2‐fold higher in G3 (E−, T+), and 1.3‐fold higher in G4 (E+, T+) (Figure 3).

IRAK2 was significantly more expressed in the G4 (E+, T+) group than in the G2 and G3 groups (*p* < 0.05). It was expressed 1.6‐fold higher in the G1 (E+, T−) group, 1.2‐fold higher in the G2 (E−, T−) group, 1.2‐fold higher in the G3 (E−, T+) group and 1.8‐fold higher in the G4 (E+, T+) group (Figure 3).

IKBKB was significantly more expressed in the G4 (E+, T+) group than in the G2 and G3 groups (*p* < 0.05). It was expressed 2.1‐fold higher in the G1 (E+, T−) group, 1.3‐fold higher in the G2 (E−, T−) group, 1.5‐fold higher in the G3 (E−, T+) group and 2.7‐fold higher in the G4 (E+, T+) group (Figure 3).

IKBKG was significantly more expressed in the G4 (E+, T+) group than in the G1 group (*p* < 0.05). It was expressed 1‐fold higher in the G1 (E+, T−) group, 1.3‐fold higher in the G2 (E−, T−) group, 1.4‐fold higher in the G3 (E−, T+) group and 2‐fold higher in the G4 (E+, T+) group (Figure 3).

BCL‐3 gene expression was significantly higher in the G3 (E−, T+) group than in all other groups. It was expressed 0.7‐fold higher in the G1 (E+, T−) group, 1.2‐fold higher in the G2 (E−, T−) group, 3.0‐fold higher in the G3 (E−, T+) group and 1.7‐fold higher in the G4 (E+, T+) group (Figure 3).

**FIGURE 4:**
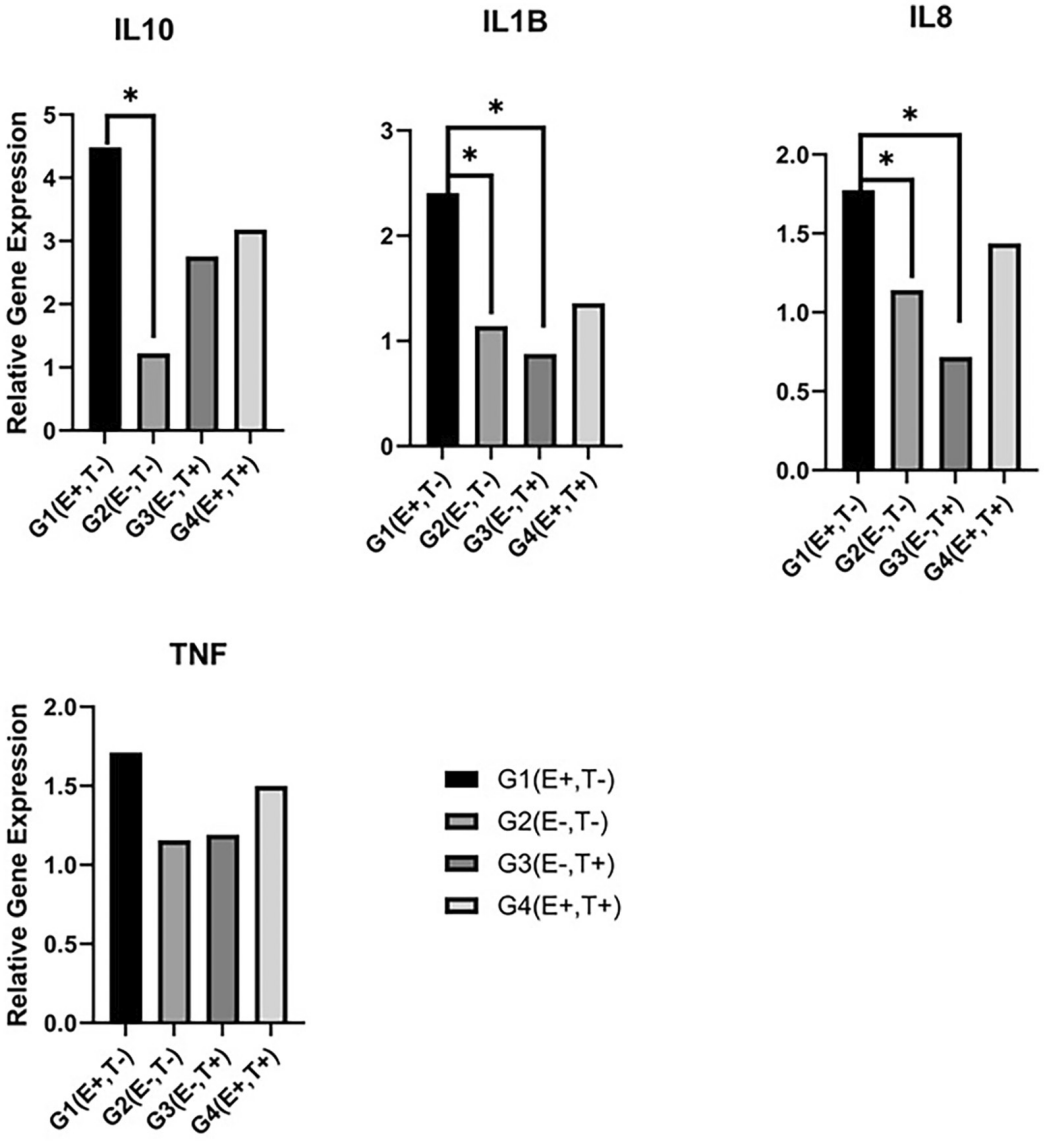
Relative gene expression levels of IL10, IL1β, TNFα, and IL8. HMGB1 gene expression level in G1 (E+, T−) group. *G1 was significantly different from other groups (*p* < 0.05).

There was no significant difference between the groups in terms of TNFα gene expression levels. β‐actin (housekeeping gene) was expressed 1.7 times higher in G1 (E+, T−), 1.2 times higher in G2 (E−, T−), 1.2 times higher in G3 (E+, T−), and 1.5 times higher in G4 (E+, T+) (Figure 4).

According to the gene expression level of IL10, it was statistically significantly more expressed in the G1 (E+, T−) group than in the G2 (E−, T−) group. It was expressed 4.5 times more in G1 (E+, T−), 1.2 times more in G2 (E−, T−), 2.8 times more in G3 (E+, T−), and 3.2 times more in G4 (E+, T+) (Figure 4).

According to the gene expression level of IL1β, it was expressed more in the G1 (E+, T−) group than in the G2 (E−, T−) and G3 (E−, T+) groups. It was expressed 2.4 times more in G1 (E+, T−), 1.1 times more in G2 (E−, T−), 0.9 times more in G3 (E+, T−), and 1.4 times more in G4 (E+, T+) (Figure 4).

According to the gene expression levels of IL8, it was expressed more in the G1 (E+, T−) group than in the G2 (E−, T−) and G3 (E−, T+) groups. It was expressed 1.8 times more in G1 (E+, T−), 1.1 times more in G2 (E−, T−), 0.7 times more in G3 (E+, T−), and 1.4 times more in G4 (E+, T+) (Figure 4).

HMGB1 is passively and actively released from cells. It is passively released from necrotic, apoptotic, and autophagic cells and actively released between cells by inflammation and immune activation. In theCNS, HMGB1 is actively released from microglia and passively from dying neurons. HMGB1 has the ability to bind to RAGE, TLR4, TLR3, TLR2, TLR1 and IL1RA. Figure 6.

## 4 DISCUSSION

Many studies have shown that *T. gondii* tachyzoites and antigens can bind to TLR4 as in HMGB1. Recently, there has been an increased focus on the diagnosis and treatment of inflammation as a cause of epilepsy, as well as drug‐resistant epilepsy and epilepsy of unknown origin. In designing this study, our hypothesis was to answer the question ‘Could *T. gondii* infection and associated inflammation be the cause of idiopathic epilepsy?’

A clinical study suggested that increased HMGB1 or TLR4 expression is associated with a higher risk and severity of epilepsy, as well as an increased likelihood of anticonvulsant drug resistance.^18^ In addition, activation of the drug‐resistant HMGB1/TLR4 pathway has also been demonstrated in surgically removed brain tissue.^17^ In their study conducted in children aged 4–14 years, Kamaşak et al. found that serum HMGB1 and TLR4 levels were significantly higher in the severe epilepsy group compared to the control group or mild epilepsy group, and in the mild epilepsy group compared to the control group. It is suggested that HMGB1 and TLR4 expression levels correlate with epilepsy severity.^38^

Neuroimmune responses lead to the production and release of active inflammatory factors such as HMGB1. Extracellular HMGB1 binds to TLR4 on the surface of neurons and glial cells, leading to seizures. Seizures cause neuronal damage, which in turn promotes the passive release of HMGB1. Proinflammatory cytokines induced by excessive release of HMGB1 also stimulate the release of more HMGB1, which plays a key role in the initiation and continuation of seizures.^18^ Experimental and clinical studies suggest that the HMGB1/TLR4 pathway is involved in epileptogenesis induced by neuroimmune inflammatory responses and contributes to the neuroinflammatory response of brain injury after epilepsy.

In a clinical study, the serum concentration of HMGB1 was negatively correlated with intelligence scores of patients, whereas it was positively correlated with seizure frequency and number of epileptiform discharges. This suggests that HMGB1 serum concentration may be involved in the initiation and progression of epilepsy or epileptic lesions and is a potential predictive factor for epilepsy prognosis.^19^ In a rat model of temporal lobe epilepsy, it has been shown that HMGB1/TLR4 is overexpressed and may induce inflammatory responses and reorganization of neuronal synaptic transmission through the p38MAPK signalling pathway.^23^

In our study, HMGB1 was expressed more than the control gene in all groups. In parallel with the literature, HMGB1 expression was also higher in the epilepsy group in our study. In many epilepsy models, HMGB1 was found to be high in both clinical and preclinical studies. In our research, whole blood samples from patients were studied. In the literature, HMGB1 has been found to be elevated in brain tissue and cerebrospinal fluid (CSF) samples obtained after epilepsy surgery, as in our study.

RAGE is a receptor that can bind to HMGB1. RAGE receptors on the cell membrane also have the ability to bind.

**FIGURE 5:**
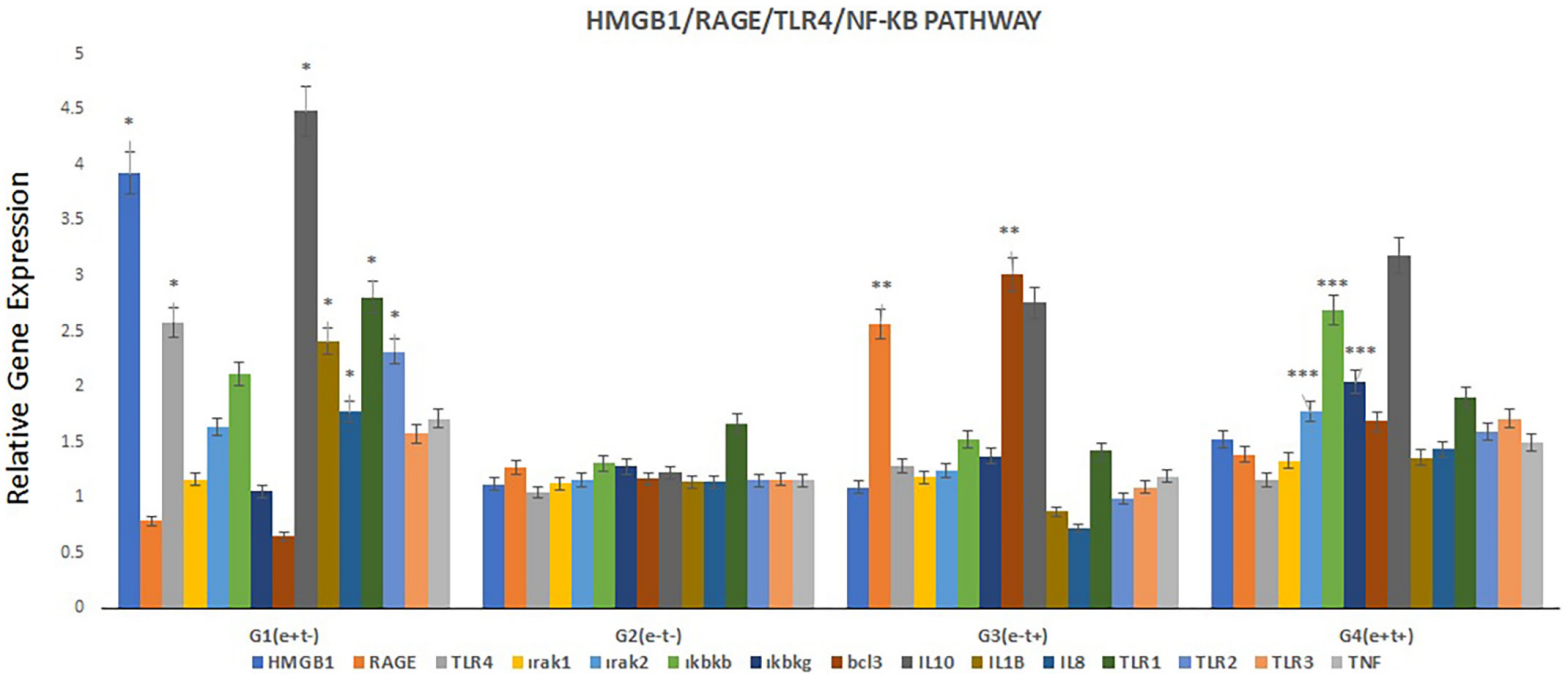
Comparison of all groups and all genes in the HMGB1/RAGE/TLR4 pathway. *G1 was significantly different from other groups (*p* < 0.05); **G3 was significantly different from other groups; ***G4 was significantly different from other groups.

HMGB1 affects neuronal excitability by increasing the extracellular glutamate concentration by inhibiting the astrocyte glutamate transporter.^29^ It has been reported that the NMDA‐NR2B receptor causes Ca^2+^ influx through activation of the HMGB1/RAGE/TLR4 signalling pathway, which in turn increases neuronal excitability and induces epileptogenesis.^39^ Figure 5.

**FIGURE 6:**
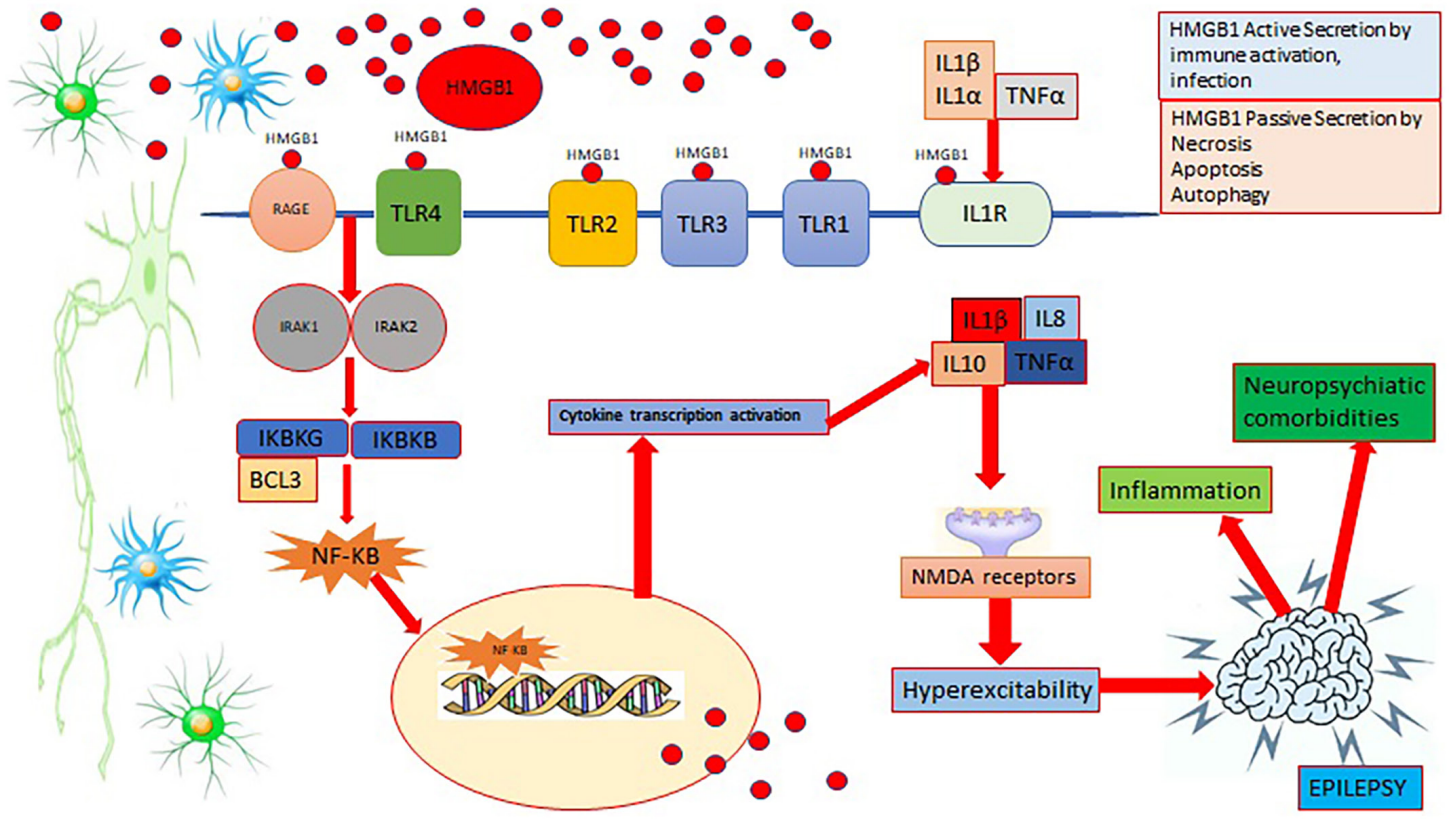
HMGB1/RAGE/TLR4/NF‐κB signalling pathway in epilepsy.

Increased RAGE expression can also lead to neuronal hyperexcitability.^40^ Seizures lead to brain cell damage and passive release of HMGB1, leading to a vicious cycle. Anti‐HMGB1 mAb may show antiepileptic effects by inhibiting the HMGB1‐TLR4 regulatory axis and reducing seizure frequency.^41^ Constitutively expressed neuronal RAGE contributes to hippocampal cornu ammonis (CA) 1 cell survival in the early phase of status epilepticus (SE) and leads to an increase in the number of neuronal cells in later stages of epileptogenesis.^42^

HMGB1 can bind to several receptors, such as RAGE, TLR2 and TLR4, for advanced glycation end‐products. After binding to TLR2, TLR4 or RAGE, HMGB1 can trigger the activation of cytokines and affect downstream inflammatory factors that play an important role in modulating neuronal excitability, which can lead to the development of epilepsy.^27^

In our study, the RAGE gene expression level was highest in the *T. gondii* + group. In the epilepsy group, however, the RAGE gene expression level was low. In the literature, the inflammatory pathway activated by HMGB1 in epilepsy does not use the RAGE receptor.

In a model of pilocarpine‐induced epilepsy, pharmacological inactivation of anti‐HMGB1 with a monoclonal antibody showed protective effects on neuronal apoptosis by reducing seizure severity and frequency and prevented epileptogenesis caused by the inhibition of HMGB1 release.^43^ Anti‐HMGB1 monoclonal antibody therapy may be a new strategy in the prevention of epileptogenesis.^44^

Anti‐HMGB1 monoclonal antibody therapy has been found to reduce electroshock‐ and pentylenetetrazole‐induced acute seizures. In TLR4‐knockout mice, however, anti‐HMGB1 monoclonal antibody did not show any anti‐seizure effect. This finding proves that the HMGB1‐TLR4 regulatory pathway contributes to epileptogenesis.^45^ The anti‐seizure effect of the anti‐HMGB1 monoclonal antibody has been shown to have sufficient potential to treat seizures in both the kainic acid model and in tissue slices from patients with medically refractory temporal lobe epilepsy.^45^

Activation of the HMGB1‐associated pathway is evident in surgically resected brain tissues from epilepsy patients. Zurolo et al. reported increased expression of HMGB1 and its downstream receptors such as TLR2, TLR4 and RAGE in pathological brain tissue from patients with focal cortical dysplasia (FCD) for the first time. IL‐1β has been shown to mediate downstream inflammatory pathways after being induced by the translocation of HMGB1 from the nucleus to the cytoplasm in glial cells.^46^

In their study, Zhang et al. found that increased translocation of HMGB1 from the nucleus to the cytoplasm has significantly increased TLR4 protein levels, cytoplasmic HMGB1, and inflammatory factors such as IL‐1β and tumour necrosis factor‐α (TNF‐α) in FCD pathological tissues compared to controls.^47^

The anti‐seizure effect of anti‐HMGB1 mAb has been found to be mediated by downstream TLR4, as TLR4−/− mice were resistant to seizure induction, and the anti‐seizure effect of anti‐HMGB1 mAb was absent in TLR4−/− mice.^48^, ^49^ This effect was also observed in brain samples from patients with refractory epilepsy.^50^, ^51^ Yang also showed that the HMGB1‐TLR4 axis promotes the development of temporal lobe epilepsy in a pilocarpine‐induced SE model in immature rats and children.^50^ In addition, activation of the IL‐1R1/TLR4 axis in neurons can increase Ca^2+^ influx through NMDA receptors, which promotes excitotoxicity and seizures.^42^, ^50^, ^52^ IL‐1β or HMGB1 may lower the seizure threshold by increasing neuronal sensitivity to NMDA, allowing the recruitment of more neurons into NMDA receptor‐mediated excitatory loops.^53^

In addition to the HMGB1‐TLR4 pathway, Iori et al. found that HMGB1 activates RAGE, which contributes to hyperexcitability and acute/chronic epilepsies as well as the pro‐seizure effects of HMGB1.^42^ However, TLR4−/− mice had much lower KA seizures while RAGE−/− mice did not have a delay in seizures, which could be explained by the fact that RAGE has a less pronounced contribution to seizures than TLR4.^42^

In our study, the TLR4 gene expression level was higher in the epilepsy group. HMGB1 binds to the TLR4 receptor in the epilepsy group and activates the inflammatory pathway. This result in our study is in line with the literature.

Parasites induce both cellular and behavioural changes in their hosts. *T. gondii* infection is associated with neurological disease and has been reported to cause behavioural changes in animals. Infected animals exhibit risky behaviour toward their predators. *T. gondii* infection in humans has been associated with behavioural and personality changes. Many parasites, such as *T. canis* and *T. gondii*, cross the blood–brain barrier and colonize the CNS, exerting effects on anxiety and control of locomotor activity.^54^, ^55^

The NF‐κB family of transcription factors is activated by many infectious and inflammatory stimuli. This family regulates the expression of many genes whose products include cytokines, chemokines, adhesion molecules and antiapoptotic factors, which are important components of the innate and adaptive immune response.^56^

TLR2 and TLR4 promote the recognition and stimulation of immune responses against *T. gondii*.^57^, ^58^ Application of *T. gondii* antigens on Ana‐1 cells was found to suppress the expression of both TLR2 and TLR4. TLR4 can use both MYD88‐ and TRIF‐dependent pathways to activate the downstream proinflammatory transcription factor NF‐κB.^59^ Downregulation of TLR4 inhibits NF‐κB signalling, which is followed by a reduction in the pro‐inflammatory cytokines TNF‐α and IL‐1β.

Activated macrophages produce nitric oxide to control *T. gondii* proliferation^60^ In mouse macrophages infected with *T. gondii*, NO production has been shown to be partially inhibited by impairment of the inducible isoform of nitric oxide synthase (iNOS).^60^ *T. gondii* antigens inhibit nitric oxide production by mouse macrophages, thereby attenuating the intracellular killing effect of macrophages on *T. gondii* and providing an effective escape mechanism.

*T. gondii* antigens from both virulent and less virulent *T. gondii* strains exhibit chemokine‐like activity, leading to a dysfunctional dendritic cell‐mediated immune response.^61^ In a study of macrophages produced from mouse bone marrow in cell culture, *T. gondii* antigens were shown to inhibit the upregulation of major histocompatibility complex (MHC) class II molecules and also to inhibit the release of TNF‐α.^62^

In a similar study, *T. gondii* antigens decreased the viability of the Ana‐1 cell line and induced apoptosis. Furthermore, the culture supernatant of *T. gondii* was shown to inhibit proliferation and induce apoptosis of human gastric cancer BGC‐823 cells.^63^

Efficient macrophages engulf and kill pathogens,^64^ but the phagocytic capacity of Ana‐1 macrophages treated with *T. gondii* antigens was found to be reduced. This could be considered a favourable strategy for the survival of the parasite.

Pro‐inflammatory cytokines such as IL‐1β, IL‐18, IL‐12, IFN‐γ and TNF‐α are critical for host resistance toward *T. gondii*,^65^, ^66^ whereas anti‐inflammatory cytokines such as IL‐10 and TGF‐β inhibit host resistance by suppressing the secretion of pro‐inflammatory cytokines.^67^, ^68^

*T. gondii*‐infected macrophages have been shown to inhibit the production of pro‐inflammatory cytokines while stimulating the expression of anti‐inflammatory cytokines. These effects create an anti‐inflammatory microenvironment for parasite replication.^65^ *T. gondii* tachyzoites inhibit the transcription factor NF‐κB and suppress the secretion of the proinflammatory cytokines TNF‐α and IL‐12.^67^

Although pathogens use various strategies to break down the host immune system, modulation of the NF‐κB pathway appears to be an important target of the immune response.^69^ The NF‐κB pathway is critical for the host immune response, but pathogens, bacteria,^70^ viruses^71^ and protozoan parasites have evolved numerous ways to block this pathway.^72^

In our study, the TLR2 gene expression level was significantly higher in the epilepsy group. These findings are in line with the literature and are accepted as one of the starting points of the inflammatory pathway in epilepsy. It is at the lowest level in the *T. gondii* group. In this study, the TLR2 gene expression level was low in *T. gondii* IgG‐positive patients. We can state that the initiation of the inflammatory signalling pathway in *T. gondii* IgG‐positive patients is not realized through TLR2.

TLR3 was mostly expressed in both the *T. gondii* positive and epilepsy groups. It was expressed at a low level in the control group and only in the *T. gondii* positive groups, while its level of expression in the epilepsy group was closest to the G4 group. Based on these results, it can be considered that epilepsy may be associated with TLR3.

When TLR1 gene expression levels were evaluated, there was no significant difference between the groups. However, TLR1 was highly expressed in the epilepsy group.

There was no significant difference in terms of IRAK1 gene expression levels between the groups. However, IRAK1 was expressed approximately 1.2 times higher than the control gene in each group.

IRAK2 was mostly expressed in the G4 group and highly expressed in the G1 epilepsy group. IRAK1 and IRAK2 are the proteins in the inflammatory pathway. These two proteins have a common effect in epilepsy and toxoplasmosis.

IKBKB and IKBKG were highly expressed in the G4 group. IKBKB was also highly expressed in the epilepsy group, while IKBKG was higher only in the *T. gondii*‐positive groups. Based on these results, we can state that epilepsy and *T. gondii* use different proteins in the HMGB1/RAGE/TLR4/NF‐κB signalling pathway.

BCL3 was expressed more in the G3 group of *T. gondii*‐positive patients than in all other groups. It can be concluded that *T. gondii* uses BCL3 in the NF‐κB pathway since it was lowest in the epilepsy group.

TNF‐α is a proinflammatory cytokine and is involved in inflammation. It was expressed more in all groups compared to the control gene, but there was no significant difference between the groups in this study.

IL‐10 is an anti‐inflammatory cytokine. It was highly expressed in all groups compared to the control gene. In our study, it was found to be most highly expressed in the epilepsy group.

IL1B is a pro‐inflammatory cytokine. Its gene expression level is the highest in epilepsy. IL1B increases even more during inflammation and increases the release of more proinflammatory cytokines.

IL8 is a pro‐inflammatory cytokine. Likewise, its gene expression is the highest in epilepsy. It exacerbates inflammation even more.

In line with the increase in the gene expression level of proinflammatory cytokines, the gene expression level of IL10, an anti‐inflammatory cytokine, was also found to increase.

When designing this study, we started with the questions ‘Could an infection be the cause of epilepsy of unknown etiology?’ and ‘Could this infection be toxoplasmosis, which is a parasite for the cell and has a very high prevalence?’ Many clinical and preclinical studies have shown that the intracellular parasite *T. gondii* is localized in the nervous system and causes many diseases. There is also a lot of evidence about epilepsy. In this research, we studied blood samples from patients with idiopathic epilepsy who received treatment, the control group (individuals with neither epilepsy nor *T. gondii* infection), *T. gondii* IgG‐positive patients, and patients with a combination of both. What we expected to see in this study was that inflammation would be higher in *T. gondii*‐positive individuals. We expected to see more seizures and epileptogenesis due to inflammation. We believed that there might be an initial factor that increases inflammation by increasing the gene expression level of proinflammatory cytokines by activating the HMGB1/TLR4/RAGE/NF‐κB pathway due to infection. However, the group with the highest activation of the HMGB1/TLR4/RAGE/NF‐κB pathway was the epilepsy group in this study. In both epilepsy and *T. gondii* positive groups, the activation of different receptors and proteins of this pathway and the suppression of inflammation by *T. gondii* resulted in less inflammation compared to the epilepsy group.

In this study, gene expression was examined in the blood samples of *T. gondii* IgG‐positive patients; our results may be attributed to the chronic nature of the infection and examination of only whole blood samples. It would be a more correct approach to studying gene expressions in blood in CSF samples. Studies on individuals with acute *T. gondii* infection may provide a better understanding of the issue.

In addition to elevated levels of HMGB1 in the brain, high levels of HMGB1 in the serum were also observed in epilepsy patients. After the onset of epilepsy, the total serum concentration of HMGB1 increases significantly and is particularly high in patients with drug‐resistant epilepsy, which may be one of the reasons for their susceptibility to recurrent seizures. Kan et al. also reported elevated serum HMGB1 concentration in patients with epilepsy and its correlation with seizure severity,^18^ suggesting that serum HMGB1 level may be a predictor of seizure severity and drug resistance in epilepsy.

### 4.1 The limitations of the study

The sample size of the study is one of our limitations. A larger number of individuals in all groups could have enabled us to obtain more reliable results. The study was conducted on blood samples. Also studying the CSF may support the results of this study. By being aware of these limitations, future studies can be designed to address these shortcomings and obtain more comprehensive results.

## 5 CONCLUSION

Epilepsy is a burden that affects various aspects of patient and family life. In addition, the burden becomes more severe by various comorbidities such as cognitive dysfunction, anxiety and depression. Therefore, there is a need to develop new biomarkers that can predict and evaluate the disease state and outcome of epilepsy treatment. There is also an urgent need to explore new treatments for epilepsy that not only delay seizure onset but also minimize associated comorbidities. In this context, there is evidence that inflammation and inflammatory processes may also cause epilepsy in addition to the previously suggested etiologies. Cytokines have been implicated in epilepsy, either as a cause of epilepsy or as a prevention or treatment strategy for epilepsy. Among these, the HMGB1/RAGE/TLR4/NF‐κB signalling pathway has been shown by clinical and preclinical studies to play a significant role in epilepsy, in addition to its role in many neurological diseases.^73^, ^74^, ^75^

Many studies have shown that the HMGB/RAGE/TLR4/NF‐κB pathway is involved in both epilepsy and toxoplasmosis. In epilepsy patients in G1, HMGB1, TLR4, IRAK2, IKBKB, IL10, IL1B and IL8 in this signalling pathway were more highly expressed, and the stimulation of excitatory mechanisms by acting through this pathway leads to seizures. The signalling pathway in epilepsy may be activated by HMGB1, TLR4 and TLR2, which is considered to increase the level of proinflammatory cytokines. In *T. gondii*, this signalling pathway was found to be activated through RAGE and BCL3.

## AUTHOR CONTRIBUTIONS

**Hayriye Soytürk:** Methodology (supporting); resources (equal); software (equal); writing – original draft (equal); writing – review and editing (equal). **Cansu Önal:** Conceptualization (equal); investigation (equal); methodology (equal); writing – review and editing (equal). **Ümit Kılıç:** Software (equal); supervision (equal); validation (equal); writing – original draft (equal); writing – review and editing (equal). **Şule Aydın Türkoğlu:** Conceptualization (equal); funding acquisition (equal); project administration (equal); software (equal); validation (equal); writing – original draft (equal). **Erol Ayaz:** Formal analysis (equal); supervision (equal); validation (equal); writing – original draft (equal).

## CONFLICT OF INTEREST STATEMENT

No conflict of interest was declared by the authors.

## Data Availability

The data that support the findings of this study are available on request from the corresponding author. The data are not publicly available due to [restrictions e.g. their containing information that could compromise the privacy of research participants].
